# Switchable modes of azulene-based single molecule–electrode coupling controlled by interfacial charge distribution†

**DOI:** 10.1039/d4sc06614f

**Published:** 2024-12-09

**Authors:** Chengyang Zhang, Yaqi Kong, Junjun Xiang, Sikang Chen, Alexei. A. Kornyshev, Jens Ulstrup, Xike Gao, Guangping Zhang, Yueqi Li, Jinghong Li

**Affiliations:** a Center for Bioanalytical Chemistry, University of Science and Technology of China Hefei 230026 China yueqili@ustc.edu.cn; b School of Physics and Electronics, Shandong Normal University Jinan 250358 China; c State Key Laboratory of Orgnometallic Chemistry, Shanghai Institute of Organic Chemistry, University of Chinese Academy of Sciences, Chinese Academy of Sciences Shanghai 200032 China; d Department of Chemistry, Imperial College London, Molecular Sciences Research Hub 82 Wood Lane London W12 0BZ UK; e Thomas Young Centre for Theory and Simulation of Materials, Imperial College London South Kensington Campus London SW7 2AZ UK; f Department of Chemistry, Technical University of Denmark 2800 Kongens Lyngby Denmark; g Department of Chemistry, Key Lab of Bioorganic Phosphorus Chemistry and Chemical Biology, Tsinghua University Beijing 100084 China

## Abstract

Molecule–electrode interactions are critical for determining transport mechanisms and device functionalities in both single-molecule electrochemistry and electronics. Crucial factors such as anchoring groups and local fields have been studied, but the role of electrolytes and interfacial charge distribution remains largely underexplored. The present research focuses on how the interfacial charge distribution in the electric double layer (EDL) controls single-molecule junctions anchored by azulene. This probe molecule is chosen for its distinct charge properties in its 5- and 7-membered condensed ring structures that impose unique sensitivity to the surrounding electric field. Using scanning tunneling microscopy break junction (STM-BJ) techniques, we systematically investigate the conductance, anchoring sites, and coupling strength of these junctions in organic liquid but non-electrolytic environments, in aqueous solution under varying ionic strengths, and across different electrode systems and potential profiles. Our results demonstrate that the conductance and molecule–electrode coupling modes can be effectively tuned through control of interfacial charge distribution, particularly by altering the ion distribution around the electrodes. Mechanical modulation experiments substantiate these trends, and theoretical calculations pinpoint ion distribution as a key driver of molecule–electrode interaction. This research introduces a novel approach to dynamic control of the azulene–electrode coupling through electrolyte manipulation, offering entirely new insight for the design of electrolyte-responsive, switchable single-molecule devices.

## Abstract 2

The coupling modes between azulene-based single molecule and electrode can be switched by the ion distribution in the electrochemical electric double layer.

## Graphical abstract

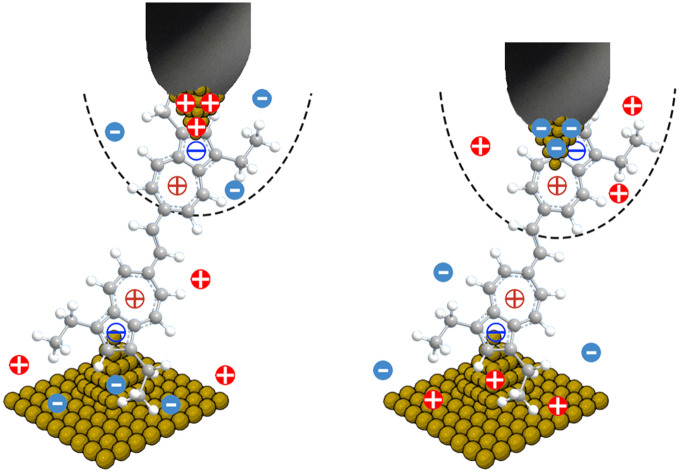

## Introduction

Understanding the interaction between a molecule and enclosing electrodes is crucial for electrical characterization at both micro- and macroscopic scales. This includes electrochemical and electronic characterization of molecular monolayers and single molecules.^1^ In molecular electronics, the coupling between a molecule and the source/drain electrodes controls transport efficiency^2,3^ and the underlying molecular mechanisms^4^ that determine the functionality of molecular devices. These interactions are affected by a range of factors, such as electrode materials,^5^ local electric fields,^6^ protonation and deprotonation,^7^ the nature of the anchoring groups,^8^ and mechanical control.^9^ Previous studies have successfully been brought to control molecule–electrode interactions at the nanoscale^10^ and even the single-molecule level,^11^ thereby controlling the electronic properties of the molecular junctions. However, while many systems involve electrolytes, studies focusing at how solution ions affect molecule–electrode coupling are scarce. Hence strategies for controlling this coupling by varying interfacial charge distribution in the electric double layer (EDL) are underexplored. By employing anchoring groups that are highly responsive to their charge environment, it becomes, however, possible to study how the interfacial charge distribution affects in detail the molecule–electrode interfaces and to develop methods to tune molecule–electrode coupling properties in response to these effects.

The present study explores how interfacial charge distribution in the EDL controls the electronic properties of single-molecule electrolyte junctions featuring azulene as a strategically chosen molecular–electrode anchoring group. The azulene molecular family, with its variable but distinct 5- and 7-membered condensed ring system carrying opposite charges offers unique sensitivity to a surrounding electric field^12^ and has shown promise as an anchor to gold electrodes *via* Au–π electronic coupling.^13,14^ Using scanning tunneling microscopy break junction (STM-BJ) techniques^15^ combined with mechanical modulation, we examined the conductance and coupling behaviour of these single-molecule junctions in liquid non-electrolytic solutions, aqueous electrolytic solutions with various ionic strength, and across different electrode systems and potential profiles. Our findings show that the molecule–electrode coupling modes and hence the conductance of the molecules can be effectively controlled by adjusting the interaction between the molecular rings and the nearby ions at the tip electrode. Theoretical calculations support that distribution of cations/anions around the electrodes is crucial in modulating the molecule–electrode coupling. Our study demonstrates the potential of tuning azulene–electrode coupling *via* electrochemical double-layer manipulation, offering a novel strategy for developing electrolyte-controlled, switchable single-molecule devices.^16–18^

## Results and discussion

### Experimental design

**Fig. 1:**
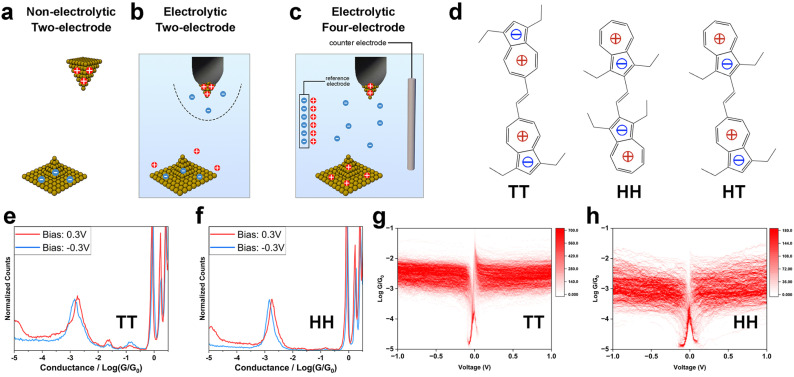
Experimental setup, molecular structures, and single-molecule electronic measurements in non-electrolyte solutions. Schematic diagrams of (a) two-electrode system in non-electrolytic solution; (b) two-electrode system in aqueous electrolytic solution; (c) four-electrode system in aqueous electrolytic solution; (d) chemical structures of TT, HH and HT; (e) one-dimensional (1D) conductance histograms of TT at ±0.3 V bias; (f) 1D conductance histograms of HH at ±0.3 V bias; (g) two-dimensional (2D) *G*–*V* histogram of TT; (h) 2D *G*–*V* histogram of HH. Singularities near 0 V are from delay of current response during the 10 Hz bias sweep. A mixed (1 : 4) solvent of tetrahydrofuran (THF) and mesitylene (TMB) was used for the non-electrolytic two-electrode system.

To study the influence of interfacial charge distribution on molecule–electrode coupling, we conducted single-molecule electronic and mechanical modulation measurements using the STM-BJ technique.^15,19,20^ In the STM-BJ measurement, we repeatedly move the STM tip electrode in and out of contact with the sample on the substrate electrode, during which single molecules bridge the two electrodes (Experimental). The recorded tip-substrate current and tip displacement reflect the conductance, mechanical stability and the length of the metal–molecule–metal junctions. Measurements under three different conditions were carried out: a two-electrode system in non-electrolytic solution (Fig. 1a); a two-electrode system in aqueous electrolyte solution (Fig. 1b); and an electrochemically controlled four-electrode system in electrolyte solution (Fig. 1c). These setups allowed us to modulate the surface charge on the electrodes and the ion distribution near the electrodes by adjusting the polarity and magnitude of tip-substrate bias, the electrochemical gate potential and the ionic strength of the solution. We examined a series of compounds terminated with condensed aromatic azulene groups on both ends, which anchored to Au electrodes *via* Au–π electronic interactions^13,14,21^ (Fig. 1d). Due to intra-molecular electron transfer, azulene's 7-membered rings are positively charged, and the 5-membered rings negatively charged, leaving the overall dipolar system sensitive to the surrounding electric field. These molecules are all in *trans*-form yet have different dipole arrangements of the azulene rings, *i.e.*, tail-to-tail (TT), head-to-head (HH) and head-to-tail (HT), where the 5-membered ring and 7-membered ring of the azulene core are defined as “head(H)” and “tail(T)”, respectively (Fig. 1d). The azulene synthesis procedure can be found in our previous report^22^).

### Static mode of molecule–electrode coupling in non-electrolytic environment

To explore how electrode surface charge affects the azulene–electrode electronic coupling, we conducted single-molecule conductance measurements using an Au tip and an Au substrate under bias voltages (*V*_bias_) of 0.1 V, 0.3 V and −0.3 V for TT, HH and HT in a mixed (1 : 4) solvent of tetrahydrofuran (THF) and mesitylene (TMB). The positive bias scenario denotes a positively charged tip and a negatively charged substrate (Fig. 1a), while a negative bias scenario denotes the opposite configuration. The surface charge density is determined by the amplitude of *V*_bias_. In these single-molecule break junction measurements, conductance plateaus signaling the formation of molecular junctions were frequently observed in conductance–distance curves for TT (Fig. S1†), HH (Fig. S2†) and HT (Fig. S3†) across all bias voltages tested. We constructed one-dimensional (1D) conductance histograms (Fig. 1e and f and S4†) from thousands of curves presented with plateaus for each compound studied at each *V*_bias_. The averaged conductance for TT showed minimal change across different *V*_bias_ (1.70 × 10^−3^*G*_0_, 1.87 × 10^−3^*G*_0_ and 1.45 × 10^−3^*G*_0_ at *V*_bias_ = 0.1 V, 0.3 V and −0.3 V). The weak bias dependence was also found for HH (1.86 × 10^−3^*G*_0_, 1.85 × 10^−3^*G*_0_ and 1.50 × 10^−3^*G*_0_ at *V*_bias_ = 0.1 V, 0.3 V and −0.3 V) and HT (1.70 × 10^−3^*G*_0_, 1.33 × 10^−3^*G*_0_ and 1.39 × 10^−3^*G*_0_ at *V*_bias_ = 0.1 V, 0.3 V and −0.3 V). These results show that, in a non-electrolytic environment, the overall electronic properties for TT, HH and HT, in which molecule–electrode coupling is an important aspect, are not significantly affected by variations in the electrode surface charge polarity and density.

We further examined the single-molecule electronic properties over a broader bias voltage range by recording current–voltage (*I*–*V*) characteristics. During the break junction measurements, the tip movement was halted upon detecting a conductance plateau indicating the formation of a single-molecule junction. We then swept the bias voltage within ±1 V at 10 Hz (Experimental). Each *I*–*V* curve was transformed into a conductance–voltage (*G*–*V*) curve by dividing the measured current by *V*_bias_. From hundreds of individual curves, we constructed two-dimensional (2D) *G*–*V* histograms for TT (Fig. 1g), HH (Fig. 1h) and HT (Fig. S5a†). The original *I*–*V* curves and histograms are shown in Fig. S5b–d.† The resulting *G*–*V* histograms show that the conductance remains largely constant for all three compounds across the entire ±1 V bias range. These findings suggest two key implications: (1) the molecule–electrode coupling is not significantly affected by *V*_bias_ within ±1 V range; (2) the conductance does not exhibit a notable dependence on energy level realignment within a ±1 V range, suggesting that the molecular frontier orbital levels (*E*_M_) are far from the Fermi levels (*E*_F_) of the electrodes to avoid detectable energy-dependent conductance shifts.

### Switchable molecule–electrode coupling modes in electrolytic solution

Next, we sought to investigate how the ionic distribution near the electrodes affects the azulene–electrode electronic coupling. Break junction measurements for TT, HH and HT in aqueous NaCl solution with various concentrations at different *V*_bias_ in both two-electrode and EC-controlled four-electrode systems were carried out (Experimental). The ion density near the electrodes can be controlled by salt concentration and electrode potentials.^1,18^ In particular, the electric field profile is highly sensitive to the electrode geometry and surface area, resulting in a substantially higher ionic density near the wax-coated tip electrode than near the substrate electrode in the two-electrode system^23,24^ (Fig. 1b). The perturbed and usually more condensed EDL at the STM tip is supported by theoretical studies^17,24–26^ and confirmed by previous experimental work.^18,24^ In the four-electrode system, a bipotentiostat was used to separately control *V*_bias_ and the potential between the tip and reference electrode. The potential between the substrate and reference electrodes is defined as *V*_EC_. To maintain comparable variation in the tunneling barrier with the two-electrode system at ±0.3 V *V*_bias_,^27,28^ we applied ±0.15 V *V*_EC_. *V*_bias_ was fixed at 0.03 V, ensuring it remained much smaller than *V*_EC_, and allowing ions of the same polarity to gather near both the tip and substrate electrodes (Fig. 1c).

**Fig. 2:**
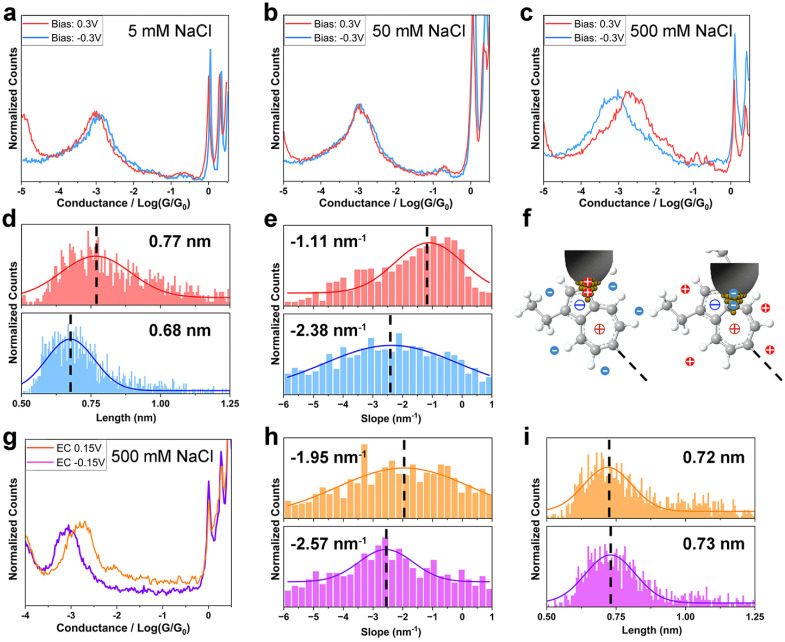
Switchable molecule–electrode coupling modes of TT in electrolyte solution. (a–c) 1D conductance histograms of TT in NaCl aqueous solution with concentrations of (a) 5 mM, (b) 50 mM, and (c) 500 mM; (d) junction length histograms of TT at bias of 0.3 V (red) and −0.3 V (blue); (e) plateau slope histograms of TT at bias of 0.3 V (red) and −0.3 V (blue); (f) proposed molecule–electrode binding configurations at positive (left) and negative (right) bias; (g–i) conductance measurements of TT at *V*_EC_ = ±0.15 V; (g) 1D conductance histograms; (h) plateau slope histograms at *V*_EC_ = 0.15 V (orange) and *V*_EC_ = −0.15 V (purple); (i) junction length histograms at *V*_EC_ = 0.15 V (orange) and *V*_EC_ = −0.15 V (purple).

We first recorded single-molecule conductance of TT in an electrolyte solution using the two-electrode system. The conductance of TT in 5 mM and 50 mM NaCl (Debye length 4.3 nm and 1.4 nm) exhibited no significant difference between positive and negative *V*_bias_ (Fig. 2a and b), similarly to the behaviour observed for non-electrolytic environment. However, at the ion strength of 500 mM (Deby length 0.43 nm), the conductance of TT is ∼3-fold higher at *V*_bias_ = 0.3 V than at *V*_bias_ = −0.3 V (2.13 × 10^−3^*G*_0_*vs.* 7.52 × 10^−4^*G*_0_, Fig. 2c and Table 1). Notably, at lower bias amplitudes (±0.1 V) in the 500 mM solution, there was no significant conductance difference when the bias polarity was reversed (Fig. S6†). These observations suggest that both the ionic strength and the *V*_bias_ amplitude are crucial factors controlling the bias-polarity dependence of single-molecule conductance of TT. This can be explained by the more effective variation in ion distribution at the azulene–electrode interface when the electrode potential difference is larger than 0.1 V and the Debye/Gouy length smaller than the molecular length; for detailed explanation of this effect see ref. 17 and 18. Previous studies^18,29,30^ have reported current rectification in single-molecule junctions in electrolyte environment due to asymmetric changes in electrode energy levels relative to molecular levels. In a limiting case, *E*_M_ is completely pinned with *E*_F_ of the substrate and all energy realignment is between the *E*_M_ and *E*_F_ of the tip. However, such rectification typically occurs when *E*_M_ is close enough to *E*_F_, similar to the pre-requisites for observable gating effects on single-molecule conductance.^24^ In contrast, the conductance of TT in non-electrolyte solution remains insensitive to energy level alignment over a broader range (±1 V, Fig. 1g), corresponding to ±0.5 V bias change in the limiting case in the two-electrode electrolyte system. The *V*_bias_ (±0.3 V) we applied in 500 mM electrolyte solution is within the “safety bias range” (±0.5 V), suggesting that the primary reason for the conductance change at opposite bias polarity is not due to energy level realignment. Instead, based on the Landauer model^31^ for charge transport, where energy level alignment and coupling strength are the two key factors, we infer that the difference in molecule–electrode coupling is the dominant factor responsible for the observed conductance change. Moreover, our *I*–*V* characterizations of TT and HH at 500 mM ionic strength show minimal rectification (Fig. S7†), in contrast to our observations from break junction measurements at static bias voltages. The lack of bias-controlled conductance switching at a fixed tip-substrate distance and under high-frequency bias variations indicates a strong dependence on junction geometry, likely requiring optimized nanogap size and structural reorganization.

**Table 1:** Conductance of TT under different testing conditions

	Positive *V*_bias_/*V*_EC_	Negative *V*_bias_/*V*_EC_
THF–TMB	1.87 × 10^−3^*G*_0_	1.45 × 10^−3^*G*_0_
5 mM NaCl	9.55 × 10^−4^*G*_0_	1.11 × 10^−3^*G*_0_
50 mM NaCl	1.10 × 10^−3^*G*_0_	1.12 × 10^−3^*G*_0_
500 mM NaCl	2.13 × 10^−3^*G*_0_	7.52 × 10^−4^*G*_0_
EC	1.60 × 10^−3^*G*_0_	8.36 × 10^−4^*G*_0_

To gain further detailed information about the molecule–electrode coupling, we constructed two-dimensional (2D) conductance/distance histograms (Fig. S8a and b†) from the conductance/distance decay curves of TT single-molecule junctions in 500 mM electrolyte solution measured at *V*_bias_ = ±0.3 V, and analyzed the junction lengths and plateau slopes from individual curves (Experimental). The junction length, *i.e.*, the width of the nanogap between the tip and substrate at junction breakdown, reflects the configuration of the junction at full molecular extension. The junction length of TT (Experimental), calculated from the tip displacement and an additional 0.5 nm snapback distance,^32^ was 0.77 nm at *V*_bias_ = 0.3 V and 0.68 nm at *V*_bias_ = −0.3 V (Fig. 2d). Both lengths are shorter than the DFT-calculated molecular length (∼1.2 nm), suggesting that the molecules bind to the electrode at a tilted angle *via* Au–π electronic interactions.^13^ Notably, the averaged junction length of TT at *V*_bias_ = 0.3 V was longer than at *V*_bias_ = −0.3 V by 0.9 Å. Assuming the contacting angle of Au–π binding remains similar for the planar azulene anchor group under both bias conditions, the elongation of the junction at 0.3 V bias likely corresponds to more distally spaced anchoring sites on the molecule.^33^ The slope of the conductance plateau (Experimental) reflects the change in junction conductance during tip retraction. When the bridged molecule's structure is rigid and its deformation negligible, the slope serves as an indicator of the dynamic molecule–electrode coupling strength during mechanical stretching,^34^ or ultimately of the alignment of the conducting MOs. At *V*_bias_ = −0.3 V, the averaged plateau slope of TT (−2.38 nm^−1^) is larger than at *V*_bias_ = 0.3 V (−1.11 nm^−1^), showing a lower stability at negative bias (Fig. 2e). This observation can be attributed to a weaker molecule–electrode coupling, which accords with the conductance trend, or a sharper dependence of the coupling strength on stretching distance.

The observed trends in conductance, junction lengths and plateau slopes of TT suggest that although the charge transport pathway through the molecule is longer at positive bias, the stronger molecule–electrode coupling maintains a higher conductance compared to negative bias. Because this switching happens only at higher electrolyte concentration (500 mM) and between larger bias contrasts (±0.3 V), we infer that interfacial ion distribution, particularly near the coated tip electrode where significant ion density changes occur, plays a critical role. Specifically, the influence of ions is the most pronounced at a distance of ∼0.3 nm—the radius of the hydrated Na^+^ or Cl^−^ ions—from the exposed surface of the tip electrode. If azulene binds to the tip electrode *via* a site near its charged center (5-membered ring for positive bias, 7-membered ring for negative bias), the surrounding cations or anions may stabilize the binding configuration by associating with the oppositely charged centers ∼0.25 nm away (anions for 7-membered ring, cations for 5-membered ring). In this system, thermodynamic stability increases when cations align near the negatively charged 5-membered ring, and anions near the positively charged 7-membered ring. Under positive/negative *V*_bias_ with anions/cations crowded near the tip electrode, the favourable coupling would thus involve the positively/negatively charged tip attaching to the region near the 5/7-membered ring, with anions/cations stabilizing the region near the oppositely charged ring (Fig. 2f). These configurations accord with the observed plateau length at different bias polarities.

Similarly, we carried out break junction measurements of the TT molecule using an electrochemically controlled four-electrode system in 500 mM electrolyte solution with *V*_bias_ = 0.03 V and *V*_EC_ = ±0.15 V. At *V*_EC_ = 0.15 V, both the tip and substrate are positively charged, while they are negatively charged at *V*_EC_ = −0.15 V. The conductance measured at *V*_EC_ = 0.15 V (1.60 × 10^−3^*G*_0_) was approximately 2 times higher than at *V*_EC_ = −0.15 V (8.36 × 10^−4^*G*_0_) (Fig. 2g and Table 1). Furthermore, we constructed the two-dimensional (2D) conductance/distance histograms (Fig. S8c and d†) and analyzed the junction lengths and plateau slopes from individual curves. The plateau slope was smaller at *V*_EC_ = 0.15 V (−1.95 nm^−1^) compared to *V*_EC_ = −0.15 V (−2.57 nm^−1^) (Fig. 2h), indicating stronger molecule–electrode coupling at positive *V*_EC_. These conductance and slope trends accord with the measurements obtained when the tip was charged in the same polarity as in the two-electrode system but are the opposite that when the substrate was similarly charged (since the tip and substrate were oppositely charged in the two-electrode system). This confirms that the charge distribution at the tip is the dominant factor influencing molecule–electrode coupling. Unlike the junction length trend in the two-electrode system, the junction length of TT at *V*_EC_ = 0.15 V and −0.15 V were comparable (0.72 nm *vs.* 0.73 nm, Fig. 2i). This can be attributed to the smaller potential difference and less dramatic change in ion distribution at the tip in the four-electrode system.

**Fig. 3:**
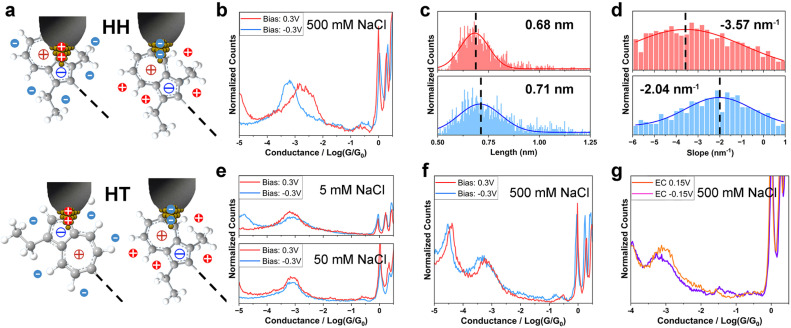
Molecule–electrode coupling of HH and HT in electrolyte solution. (a) Proposed molecule–electrode binding configurations for HH (upper) and HT (lower) in two-electrode electrolyte solution; (b) 1D conductance histograms of HH in 500 mM NaCl aqueous solution at ±0.3 V bias; (c) junction length histograms of HH at ±0.3 V bias; (d) plateau slope histograms of HH at ±0.3 V bias; (e and f) 1D conductance histograms of HT in 5 mM, 50 mM (e) and 500 mM (f) NaCl aqueous solution at ±0.3 V; (g) 1D conductance histograms of HT at *V*_EC_ = ±0.15 V.

In a parallel investigation of HH in electrolyte environment, we recorded the single-molecule conductance and analyzed the junction length and plateau slope of HH (Fig. 3a). No apparent difference in conductance between the positive and the negative *V*_bias_ (±0.1 V, ±0.3 V) in 5 mM and 50 mM electrolytic solutions was observed, nor between the positive and the negative *V*_EC_ (±0.15 V) at 500 mM ion strength (Fig. S9†), implying the need for a more pronounced variation in EDL for tuning the coupling of HH. Conductance differences only became significant at higher electrolyte concentration (500 mM) and at larger bias voltage (*V*_bias_ = ±0.3 V, with 1.71 × 10^−3^*G*_0_ at 0.3 V v.s. 6.24 × 10^−4^*G*_0_ at −0.3 V, Fig. 3b and Table 2). 2D conductance histograms (Fig. S10†), plateau length and junction length were also constructed for HH. In contrast with TT, the plateau length of HH was relatively shorter at positive bias compared to negative bias (0.68 nm *vs.* 0.71 nm, Fig. 3c), suggesting that the anchoring sites are more closely located at positive bias. In HH, the two azulene fragments are aligned head-to-head, with the 5-membered rings positioned nearer the center of the molecule. The observed trend supports our view of EDL-controlled binding configurations, where a positively charged tip attaches to a region near the 5-membered ring, while anions stabilize the area closer to the 7-membered ring. The plateau slope of HH was larger at 0.3 V bias than at −0.3 V bias (−3.57 nm^−1^*vs.* −2.04 nm^−1^, Fig. 3d). Notably, both TT and HH showed larger slope when the junction length was shorter, suggesting weaker junction stability at proximal binding sites (closer from the molecule's center). A possible explanation is that, under stretching, a contact shift from a proximal site to a more distant, oppositely charged site changes the interfacial charge interaction with the electrode and ions from attractive to repulsive, accelerating the breakdown of the binding.

Conductance of HH and HT under different testing conditions

**Table:** 

HH	Positive *V*_bias_/*V*_EC_	Negative *V*_bias_/*V*_EC_
THF–TMB	1.85 × 10^−3^*G*_0_	1.50 × 10^−3^*G*_0_
5 mM NaCl	9.44 × 10^−4^*G*_0_	8.82 × 10^−4^*G*_0_
50 mM NaCl	9.52 × 10^−4^*G*_0_	7.58 × 10^−4^*G*_0_
500 mM NaCl	1.71 × 10^−3^*G*_0_	6.24 × 10^−4^*G*_0_
EC	1.36 × 10^−3^*G*_0_	1.76 × 10^−3^*G*_0_

**Table:** 

HT	Positive *V*_bias_/*V*_EC_	Negative *V*_bias_/*V*_EC_
THF–TMB	1.33 × 10^−3^*G*_0_	1.39 × 10^−3^*G*_0_
5 mM NaCl	8.12 × 10^−4^*G*_0_	9.08 × 10^−4^*G*_0_
50 mM NaCl	9.10 × 10^−4^*G*_0_	9.32 × 10^−4^*G*_0_
500 mM NaCl	9.63 × 10^−4^*G*_0_	7.15 × 10^−4^*G*_0_
EC	8.39 × 10^−4^*G*_0_	6.82 × 10^−4^*G*_0_

We finally measured the single-molecule conductance of HT in both two- and four-electrode systems (Fig. 3e–g and Table 2). Across all measurement conditions, the conductance showed only weak dependence on the electrolyte concentration, bias voltage, and electrochemical potential. This behaviour can be attributed to the averaging effect of HT molecules bridging between the tip and substrate in two possible orientations (Fig. 3a, lower panel). The absence of switching for HT further supports our explanation that the interaction between the positively/negatively charged regions and the surrounding anions/cations is a crucial factor affecting the molecule–electrode coupling mode for TT and HH molecules.

### Mechanical modulation of molecule–electrode coupling

To further validate the preferred tip anchoring sites on the azulene structure, we imposed mechanical modulation on the TT, HH and HT single-molecule junctions. Upon detecting a plateau indicative of single-molecule junction formation during the break-junction measurements, we paused the tip retraction and applied a periodic triangular voltage modulation (±0.02 V) to the *z*-axis piezo. This modulation caused the tip to move towards and away from the sample by ±0.24 nm at a constant speed and a frequency of 12.5 Hz (Experimental, Fig. S11†). Conductance variations at different tip positions indicated the relative locations of preferred tip-molecule binding sites. Statistical analysis of hundreds of conductance curves showed that, at positive bias, TT molecules tend to bind to the tip at a more distal anchoring site (farther from the molecule's center), while at negative bias, binding occurs at a proximal site. For HH molecules, the trend reverses, with a smaller bias-dependent difference in binding sites. These findings align with our interpretations of EDL-regulated, switchable molecule–electrode coupling modes for TT and HH molecules based on STM-BJ experiments.

### Theoretical calculations

**Fig. 4:**
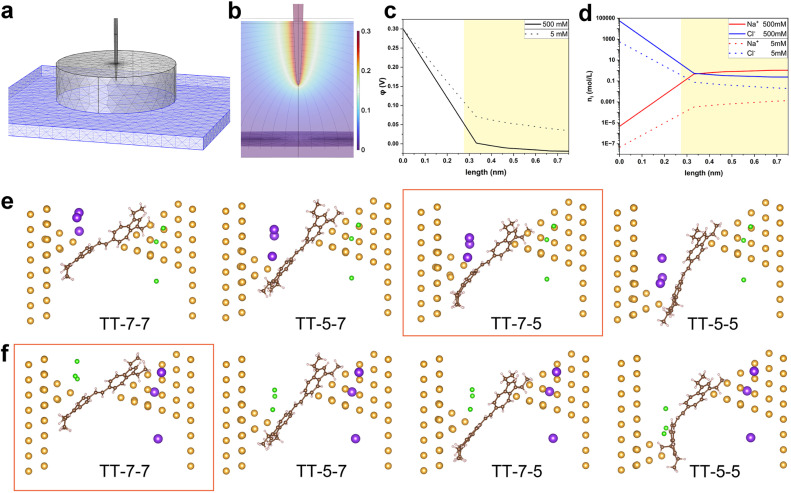
Theoretical calculations of the interfacial charge distribution and single-molecule junction geometry in electrolyte environment. (a) The model built for COMSOL simulation of (b) the electric fields and the interfacial charge distribution, (c) potential profile, and (d) cation and anion density profiles on the perpendicular line extending from the tip to the substrate at 500 mM and 5 mM electrolyte concentration and 0.3 V bias. The yellow shades highlight the regime with realistic concentration, where the ions are allowed to approach; (e and f) the optimized molecule–electrode binding geometries of TT in 500 mM NaCl electrolyte solution under (e) 0.3 V and (f) −0.3 V. The spheres in gold, brown, light coral, purple, and green represent Au, C, H, Na^+^, and Cl^−^, respectively. The left electrode represents the substrate and the right electrode the tip. The red frame marks the geometry with the lowest energy under the given bias. In the geometric optimizations, the GGA-PBE functional is used, and a basis set of SZP for Au while DZP for C, H, Na^+^, and Cl^−^ is employed. DFT-D3 semiempirical dispersion correction is applied to more accurately describe the Au–π interaction.

To further elucidate the experimental observations, theoretical simulations by COMSOL Multiphysics and DFT calculations were carried out. First, we simulated the interfacial charge distribution at the molecule–electrode interface under electrolytic conditions. COMSOL Multiphysics was used to simulate the electric potential and ion density profiles near the STM tip electrode. A continuum model was employed to simulate the electrode and electrolyte system. We modeled the system with a paraboloidally shaped Au tip (1.41 Å radius of curvature at the top) and a flat Au substrate positioned 1.5 nm away, both electrodes immersed in a continuum aqueous solution of 5 mM and 500 mM NaCl simulated by ‘Transport of Diluted Species’ model (Fig. 4a). The tip was partially submerged into the electrolyte by 1.5 nm to simulate the experimental condition where the tip was coated with Apiezon wax. The bias voltage of 0.1 V and 0.3 V between the tip and substrate were applied. The Nernst–Plank equation was used to calculate the electric field (Fig. 4b) and electric potential distribution, particularly along a perpendicular line from the tip apex to the substrate. The most dramatic field and potential change happens near the apex of the tip, where the EDL should be notably denser than near the substrate.^35^ To account for the finite size of hydrated cations and anions, we applied Stern correction to the potential profile (Fig. 4c and S12a†) and calculated the cation/anion density profiles (Fig. 4d and S12b†) based on the Poisson–Boltzmann equation. At the electrode–solution interface, the number of cations and anion concentrations are apparently higher at increased bulk ionic strength and greater bias voltage, which is expected. From these density profiles, we extracted the maximum possible cation and anion concentrations at a distance of 0.275 nm, corresponding to the diameter of a water molecule and the closest distance a cation or anion can approach the electrode surface. At 500 mM electrolyte concentration and 0.3 V bias, the calculated maximum anion concentration near the tip electrode was 4.3 M, while the cation concentration was 0.058 M. Our previous theoretical study^17^ suggests that larger ions reduce the electrolytically controlled rectification effect, due to their influence on ion distribution—specifically, larger ions result in a thicker double layer, particularly at increased bias voltage due to the so-called excluded volume or crowding effect.^36^ The effective size of ions includes their tightly bound first hydration shell, which suggests the chosen electrolyte worked as expected. However, the trend in ion size warrants further investigation, along with the potential for more complex effects on electrostatic potential and ion distributions in aqueous solutions.^37^

The most favourable molecule–electrode coupling modes as well as the charge transport properties of TT and HH single-molecule junctions were investigated next. For this, the bare TT and HH molecules were geometrically optimized using density functional theory (DFT) at the B3LYP/6-311g(d) level in the Gaussian 16 package.^38^ From the optimized structures shown in Fig. S13,† both TT and HH are distorted between the two planar azulene terminators. The distortion angle is about 35° and 57° for TT and HH, respectively. This accords with previous experimental observations.^22^ With the optimized bare TT and HH molecules, electrode-single molecule–electrode junctions were subsequently constructed. The tip electrode (the right electrode in Fig. S14†) was modelled with a large pyramidal Au cluster while a much smaller cluster was left on the substrate electrode (the left electrode in Fig. S14†) to simulate the experimental process of repeatedly moving the tip electrode in and out of contact with the substrate electrode. To mimic the change in the ability of donating or accepting electrons for the biased electrodes, the tip and substrate electrodes are oppositely “doped” by changing the ion charge of individual atoms in the subsequent geometry relaxations of single-molecule junctions. As demonstrated in Fig. S14, S15, Tables S1 and S2,† the TT molecule in non-electrolytic environment is preferentially anchored on the tip and substrate electrodes *via* the 7-membered rings, while HH is preferentially anchored *via* the 5-membered rings at both 0.3 V and −0.3 V. That is to say, proximal anchoring sites for both TT and HH are energetically favourable, and the polarity of *V*_bias_ does not substantially change the anchoring sites of azulene groups.

500 mM concentration of NaCl aqueous solution corresponds to 3 Na^+^ and 3 Cl^−^ in the cavity of the constructed single-molecule junctions, which were posed at ∼0.3 nm away from the tip and substrate electrodes as this is the radius of the hydrated Na^+^ or Cl^−^ ions as noted. As shown in Fig. 4e, f, and S16† as well as in Tables 3 and S3,† the anchoring site of azulene in TT and HH molecules on the substrate electrode in electrolytic solution remains the same as in non-electrolyte solution.

**Table 3:** The relative energy (in eV) of single-molecule TT junction calculated from each optimized molecule–electrode binding geometry in 500 mM electrolyte solution shown in Fig. 4e and f

*V*_bias_	TT-7-7	TT-5-7	TT-7-5	TT-5-5
0.3 V	0.02	2.31	0	2.28
−0.3 V	0	1.55	0.39	0.50

However, the anchoring site of azulene on the tip electrode is highly dependent on the bias polarity independently of the specific compounds (TT or HH). More specifically, both TT and HH are preferentially anchored *via* the 5-membered ring on the tip electrode at 0.3 V but *via* the 7-membered ring at −0.3 V. The theoretical result regarding the bias polarity dependent anchoring site of the azulene group in TT and HH molecules on the tip electrode accords well with the experimental observations of the bias polarity dependent junction length shown in Fig. 2d and 3c. Moreover, the above theoretical results for non-electrolytic and electrolytic conditions suggest that the molecule–electrode coupling mode of the azulene group on the tip electrode under electrolytic condition is fundamentally determined by the bias polarity dependent ion distributions.

To further confirm this conclusion, electron transport properties of the constructed single-molecule junctions were addressed by using the state-of-the-art Nonequilibrium Green's function (NEGF) method^39^ in combination with DFT implemented in the QuantumATK package.^40,41^ The transmission spectra for TT and HH single-molecule junctions with energy favourable conformations at ±0.3 V are shown in Fig. S17.† It is found that the carriers in both TT and HH single-molecule junctions are dominated by electrons, rather than holes. That is, the transmission peaks closely above the *E*_F_ for TT and HH single-molecule junctions play a key role in determining their conductance. A more detailed analysis of molecular orbitals for TT and HH bare molecules (Fig. S18† for TT as an example) and their molecular projected self-consistent Hamiltonian (MPSH) states in the single-molecule junctions (Fig. S19 and S21–S23†) suggest that the transmission peaks primarily originate from the LUMOs of the bare molecules (*e.g.* Fig. S20† for TT in TT-7-5 single-molecule junction). It should be noted that these transmission peaks are expected to locate much farther from *E*_F_ according to the experimental observation than the energy-dependent conductance shifts for both TT and HH are not obvious. This discrepancy in energy level alignment can be attributed to (1) the deficiency of DFT in accurately describing the band gap of semiconductors and molecules and (2) the effect of solvent on the energy level alignments not considered here. However, the theoretical calculations here still provide qualitative support for the experimental observations. The calculated conductance for the single-molecule junction with TT-7-5 conformation at 0.3 V is 0.51 *G*_0_ which is larger than that of 0.08 *G*_0_ for the TT-7-7 junction conformation at −0.3 V. Meanwhile, the calculated conductance for the single-molecule HH-5-5 junction at 0.3 V is 0.23 *G*_0_ and much larger than 0.01 *G*_0_ for the HH-5-7 junction at −0.3 V. The effect of anchoring site altering of azulene on the tip electrode induced by changing the bias polarity on the conductance of TT and HH single-molecule junctions thus accords qualitatively with the experimental measurements.

To gain further insight into the nature of Au-azulene electronic coupling, we examined the spatial distributions of the primary conduction orbitals and the changes in charge distribution of TT and HH molecules in the single-molecule junctions. Previous studies have shown that Au–π electronic interactions are driven by electrostatic and van der Waals forces, and often accompanied by moderate orbital hybridization between the π-system and the metal surface.^14^ Our Bader charge analysis^42^ of isolated TT and HH molecules revealed substantial charge polarization with negative charge on the 5-membered ring and positive charge on the 7-membered ring, promoting molecule–electrode binding *via* electrostatic interactions. Though there are variations in the amount of net charge carried by the 5- and 7-membered rings after single-molecule junctions formed and the existence of the surrounding solution environments as well as external biases, the polarities of the net charges in the two rings at the tip side are always the same, suggesting robustness of the molecule–electrode electrostatic interactions. On the other hand, the MPSH orbitals contributing to the conduction displayed pronounced hybridization between the molecule's LUMO and the electrode surface states (Fig. S19–S23†), confirming the presence of evident molecule–electrode coupling. This significant hybridization likely arises from notable electrostatic interactions and aligns with the robust binding events observed in this and previous studies.^13^ It should be noted that comparing the binding sites of TT and HH molecules in non-electrolyte and electrolyte environments under varying biases, the change of binding sites in electrolytic environment is attributed to the fundamental changes in electrostatic interactions between molecules and surrounding ions, the distributions of which are determined by the bias polarity.

## Experimental

### Single-molecule electronic measurements

Single-molecule break junction experiments were carried out using a scanning tunneling microscope (Agilent 5500). The STM tip was freshly prepared by cutting a gold wire (0.25 mm, 99.999%, Thermo Scientific) and coating with Apiezon wax if used in electrolyte environment. The non-electrolytic solution was prepared by dissolving the target compound in a mixed solvent of tetrahydrofuran (99.9%, Sigma-Aldrich) and mesitylene (TMB, 98%, Aladin) at a ratio of 1 : 4 (for good solubility). The electrolyte solutions were prepared by dissolving the target compound in NaCl (Sigma-Aldrich, 99%) aqueous solution. The solution was introduced into a liquid cell on a gold substrate (approximately 160 nm thick, deposited on mica (Ted Pella) under ultrahigh vacuum). The four-electrode system consists of two working electrodes (the tip and substrate), a quasi-reference electrode (silver wire) and a counter electrode (platinum coil), of which the potentials between the tip and substrate and between the tip and reference electrode are separately controlled by a built-in bipotentiostat. During the break junction measurements, the STM tip was continuously moved toward and away from the substrate following a previously reported method.^15^ The current measurements were recorded at a sampling frequency of 10 kHz. The current–voltage characteristics were recorded by stopping the tip movement upon detection of a conductance plateau and ramping the bias voltage in a triangular wave from the initial 0.1 V bias to 1 V, −1 V and back to 0.1 V with a frequency of 10 Hz.

We obtained the one-dimensional (1D) conductance histograms from thousands of individual conductance–distance curves and carried out Gaussian fittings on the conductance peaks, from which we determined the peak positions and standard deviations (*σ*). To accurately compute the junction length and plateau slope, curves that did not demonstrate junction formation were excluded. Junction length was calculated from the transition point of the Au–Au quantum contact breakdown (*G*_0_) to the plateau breakdown (*G* − 3*σ*) added by 0.5 nm Au–Au snapback distance^32^ for each curve. For the plateau slope analysis, we utilized a consistent, narrower range from (*G* + *σ*) to (*G* − *σ*) to minimize errors caused by sharp declines before and after the plateau in the curves.

### Mechanical modulation

The mechanical modulation^43^ was performed based on the STM-BJ measurements.^44^ Upon detecting a conductance plateau signalling the formation of a single-molecule junction, we halted the tip movement and applied a repeated triangular wave on the piezo voltage with a ±0.02 V amplitude and 12.5 Hz frequency, introducing a periodic oscillation of ±0.24 nm of the tip. The recorded conductance during the tip modulation was normalized to the initial conductance when the junction was first detected, resulting in the relative variation of conductance with modulation. We repeated the measurement hundreds of times and stacked the individual curves together to facilitate observation of the general trends.

## Conclusions

Using a series of azulene-based probe molecules, in this study, we explored how the electric double layer in single-molecule electrolyte junctions influences interactions between the molecule and the enclosing electrodes. Azulene-based molecules were selected for their distinct intramolecular charge distributions, making them effective probes for electrostatic mapping within electrolyte junctions. These discrete charge distributions significantly impact the arrangement of surrounding electrolyte ions near the electrodes. The strong electrostatic interactions between the dipolar, multiply charged azulene molecules and the ionic environment have enabled detailed mapping of the ionic distribution and electrostatic potential within the junction. Our methodology combines single-molecule conductance measurements, mechanical modulation experiments, and theoretical simulation and calculations in aqueous electrolytes, with non-electrolytic solutions as a reference, across various electrode systems. Notably, we demonstrated that the electrolyte's cations and anions play a critical role in determining both the molecule–electrode coupling site and the coupling strength. The key outcomes of our work include achieving an exceptional level of structural and dynamic controlling of the electrolyte junction, made possible by our advanced molecular probe design. In addition, the detailed characterization of electrolyte effects underscores their potential towards the design and operation of switchable, “smart” single-molecule devices controlled *via* electrolytic environments.

## Data availability

The data supporting this article have been included as part of the main text and the ESI.†

## Author contributions

Chengyang Zhang, Sikang Chen, Yueqi Li and Xike Gao designed and performed the experimental measurements. Yaqi Kong and Guangping Zhang performed the theoretical calculations. Junjun Xiang and Xike Gao synthesized all the molecules. Chengyang Zhang, Yaqi Kong, Alexei. A. Kornyshev, Jens Ulstrup, Xike Gao, Guangping Zhang, Yueqi Li and Jinghong Li analyzed the data and wrote the manuscript. Chengyang Zhang, Yaqi Kong and Junjun Xiang contributed equally to this paper.

## Conflicts of interest

The authors declare no competing interests.

## Supplementary Material

SC-016-D4SC06614F-s001
