# Supplementary material for: Switchable modes of azulene-based single molecule–electrode coupling controlled by interfacial charge distribution
Source: Chem Sci. 2024 Dec 9;16(3):1353–63. doi: 10.1039/d4sc06614f (PMC11653075; doi:10.1039/d4sc06614f)
Supplement: SC-016-D4SC06614F-s001 [file SC-016-D4SC06614F-s001.pdf]

Supplementary Information (SI) for Chemical Science.
This journal is © The Royal Society of Chemistry 2024

Supporting Information

Switchable modes of azulene-based single molecule-electrode coupling controlled by interfacial charge distribution

Chengyang Zhang[#a], Yaqi Kong[#b], Junjun Xiang[#c], Sikang Chen[a], Alexei. A. Kornyshev[d], Jens Ulstrup[e], Xike Gao[*c], Guangping Zhang[*b], Yueqi Li[*a], Jinghong Li[*a]

[*] To whom correspondence may be addressed.
Emails:

Yueqi Li: yueqili@ustc.edu.cn

Guangping Zhang: zhangguangping@sdnu.edu.cn

Xike Gao: gaoxk@mail.sioc.ac.cn

Jinghong Li: jhli@mail.tsinghua.edu.cn

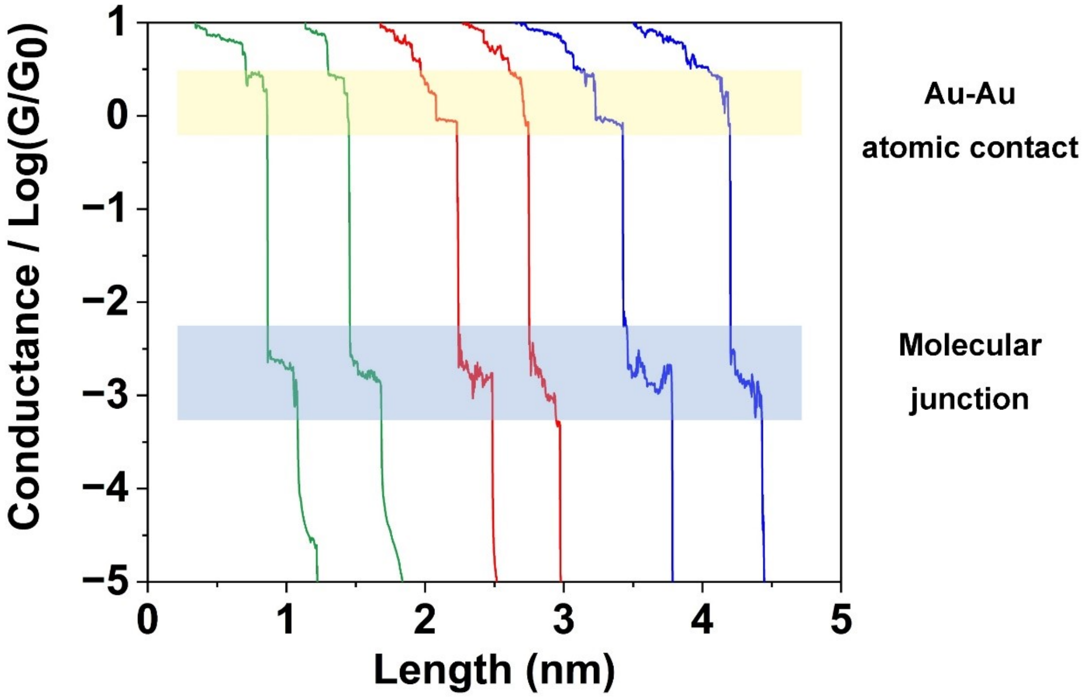

Figure S1. Conductance-distance curves for TT at bias of 0.1 V (green), 0.3 V (red) and -0.3 V (blue).

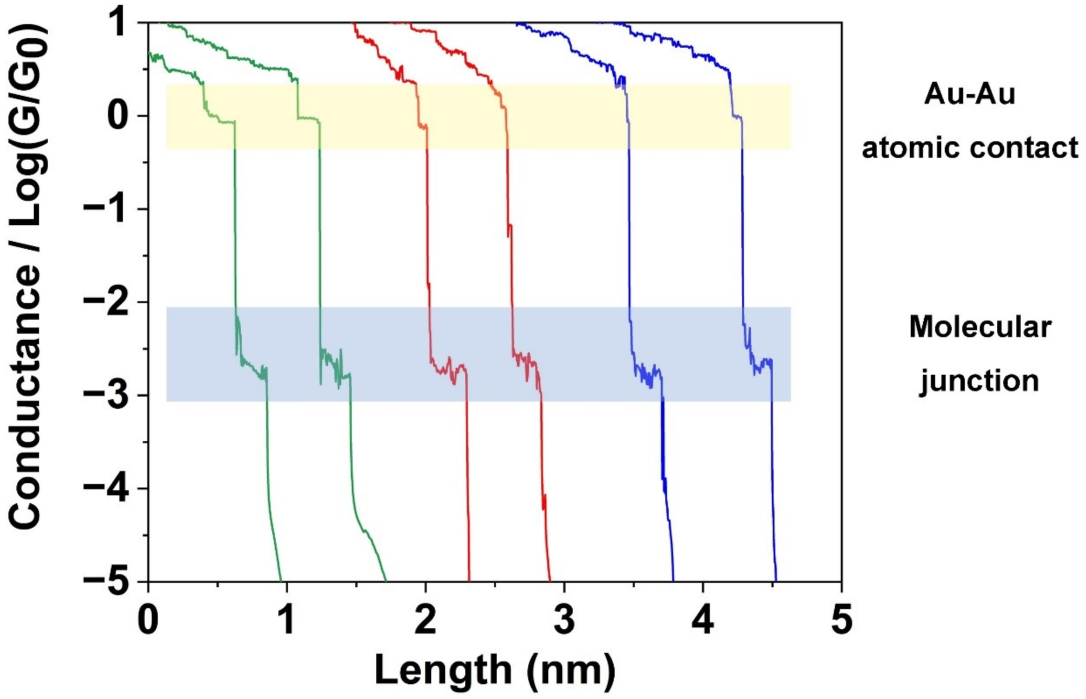

Figure S2. Conductance-distance curves for HH at bias of 0.1 V (green), 0.3 V (red) and -0.3 V (blue).

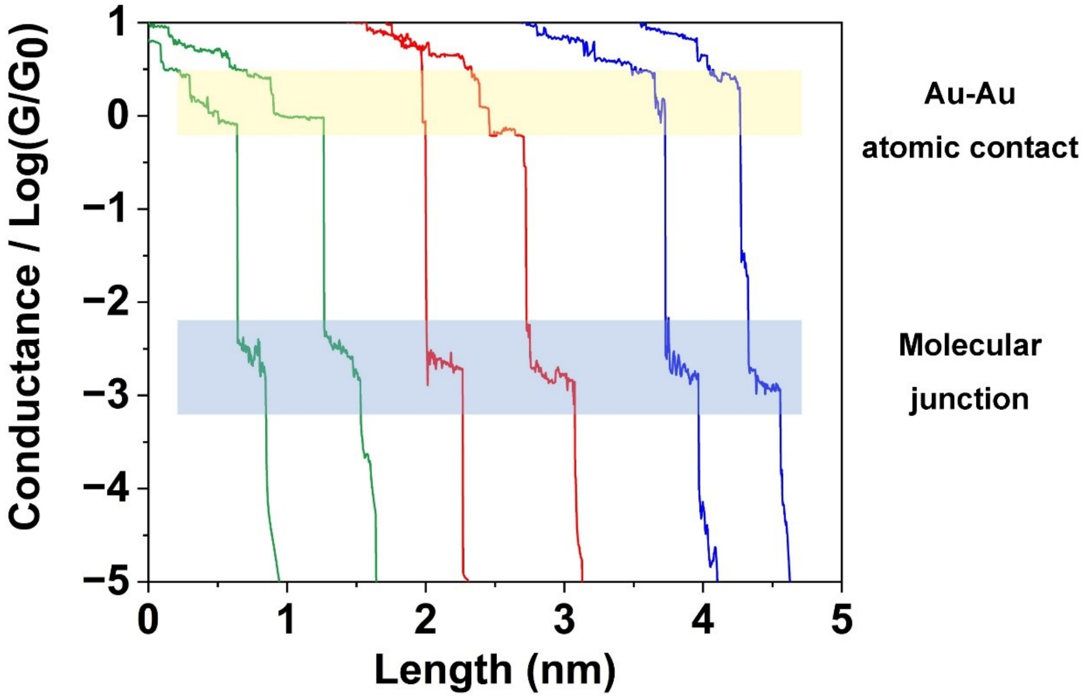

Figure S3. Conductance-distance curves for HT at bias of 0.1 V (green), 0.3 V (red) and -0.3 V (blue).

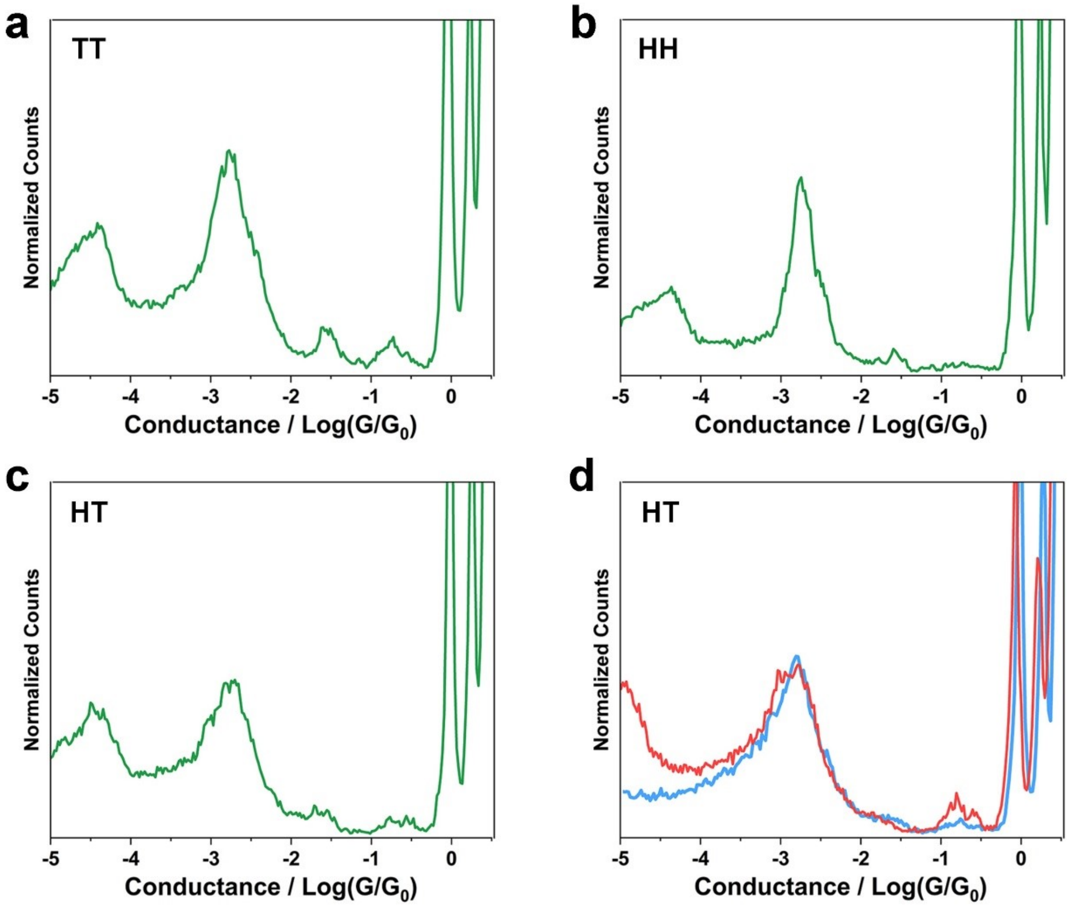

Figure S4. One-dimensional conductance histograms of TT, HH and HT. a-c) 1D histograms of TT (a), HH (b) and HT (c) at 0.1 V bias; d) 1D histogram of HT at ±0.3 V bias. The sharp peaks at and above G_0 are from atomic Au-Au contact.

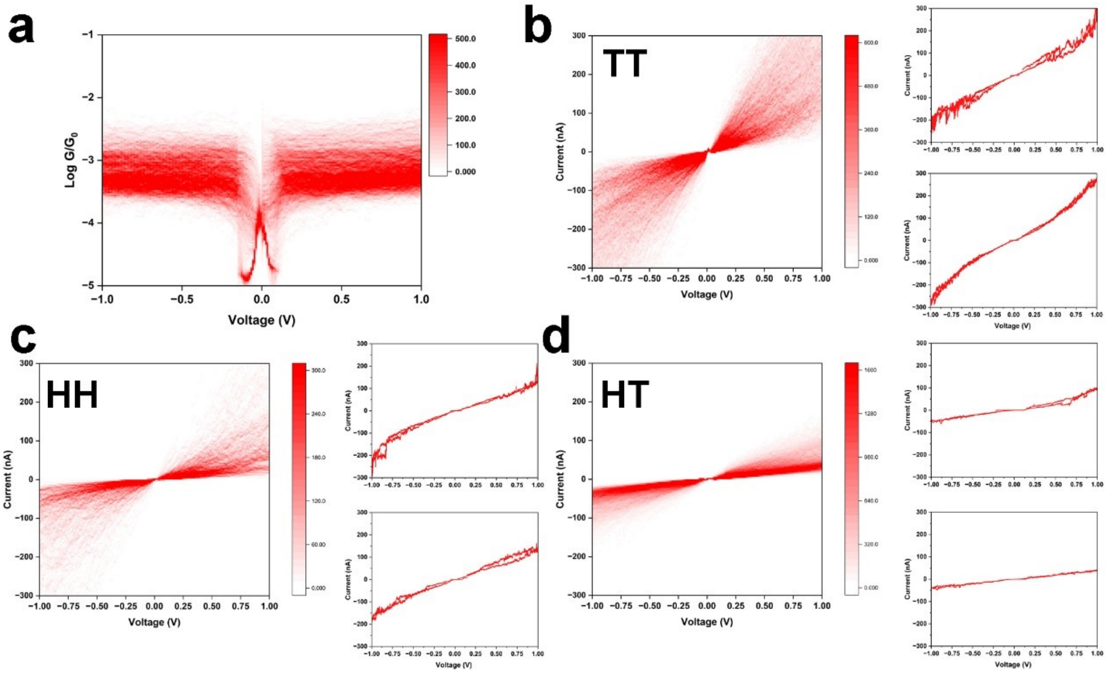

Figure S5. Two-dimensional *G-V* and *I-V* histograms. a) *G-V* histogram for HT; b-d) *I-V* histograms for TT, HH and HT, single *I-V* curves are shown on the right.

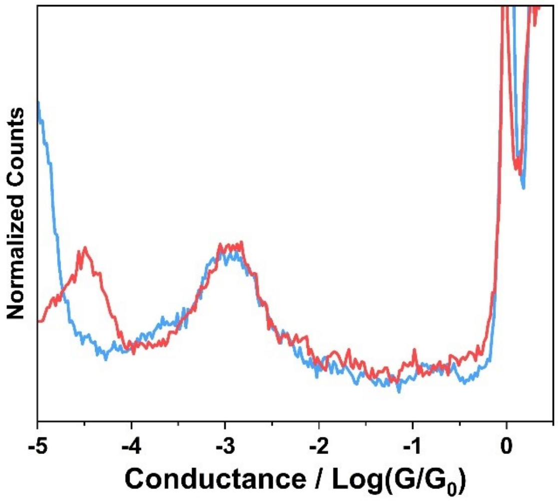

Figure S6. One-dimensional conductance histogram of TT in NaCl aqueous solution with concentrations of 500 mM at 0.1 V (red) and -0.1 V (blue).

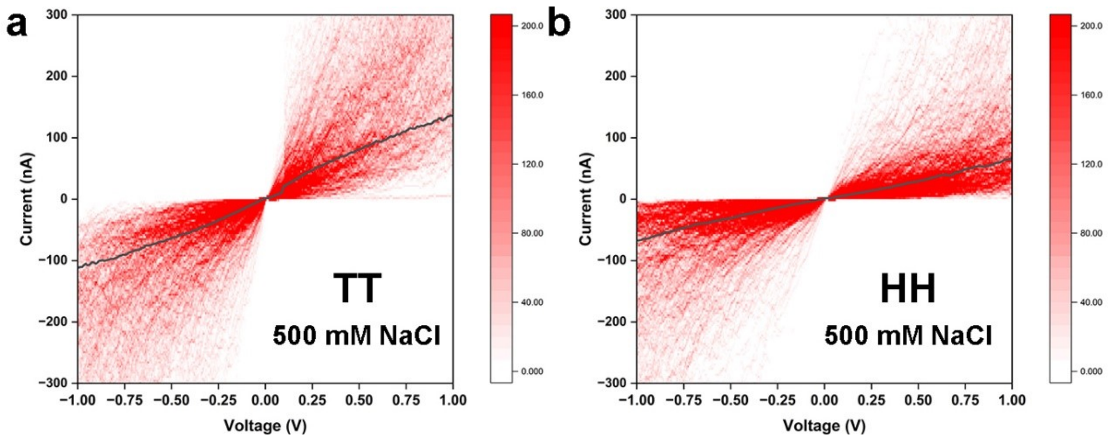

Figure S7. Two-dimensional *I-V* histograms of TT and HH in 500 mM NaCl aqueous solution. a) *I-V* histogram for TT; b) *I-V* histogram for HH.

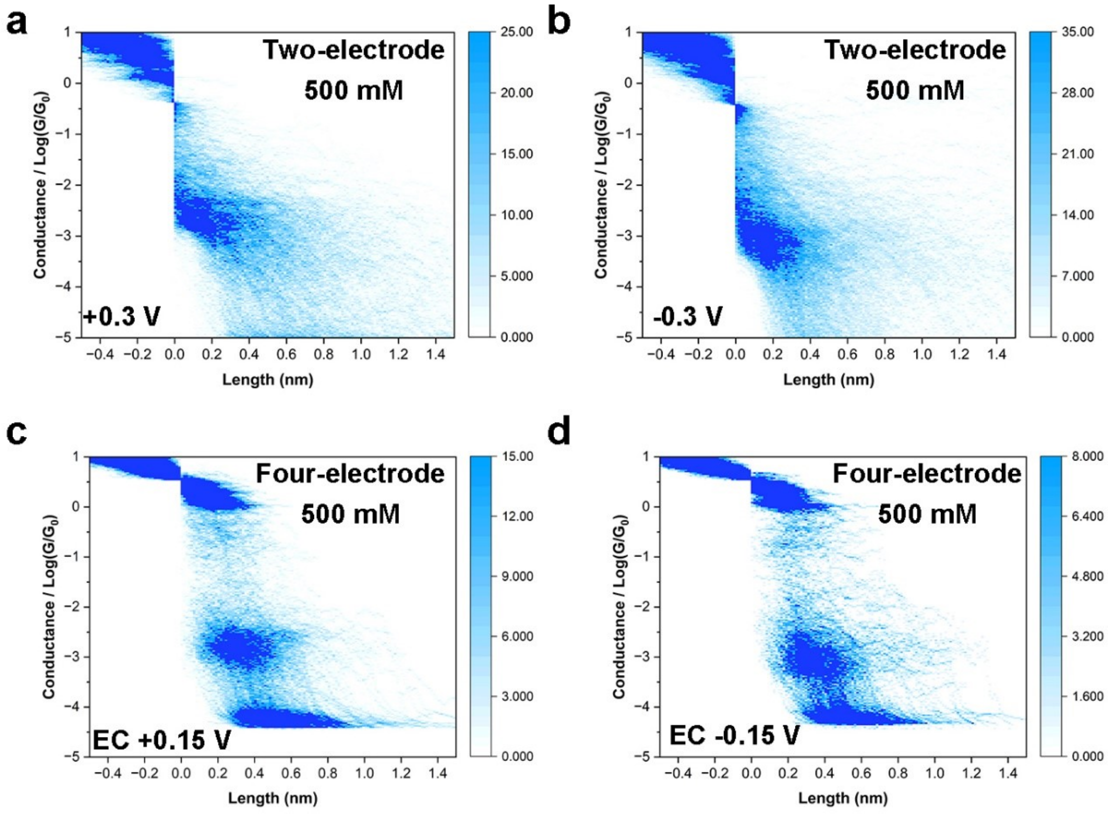

Figure S8. Two-dimensional conductance/distance histograms of TT. a) TT at bias of 0.3 V in two-electrode system in 500 mM aqueous NaCl solution; b) TT at bias of -0.3 V in two-electrode system; c) TT at V_{EC} of +0.15 V in four-electrode system; d) TT at V_{EC} of -0.15 V in four-electrode system.

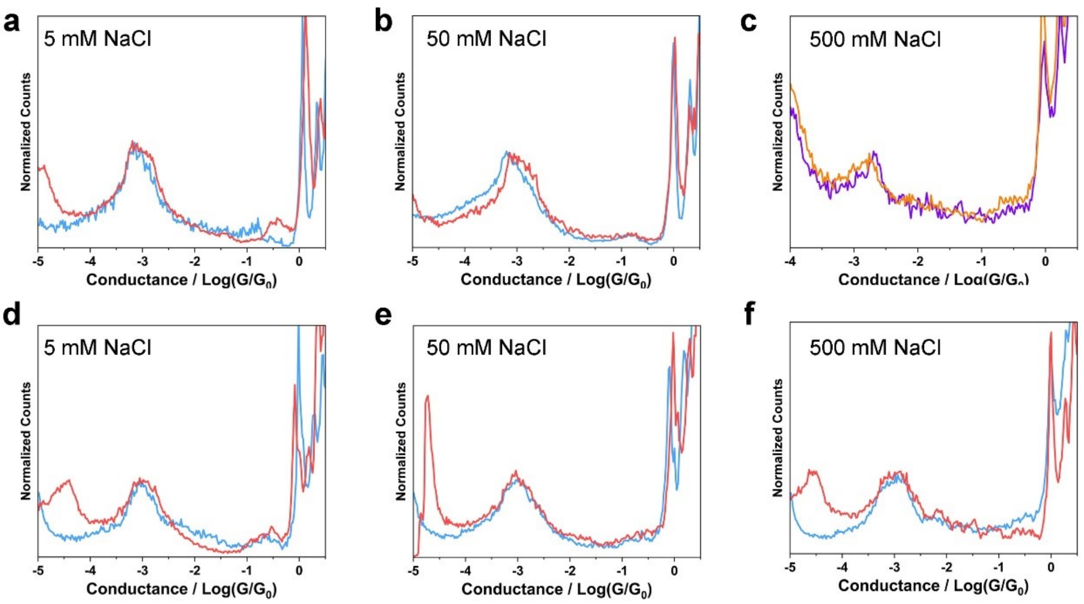

Figure S9. One-dimensional conductance histograms of HH. ±0.3 V_{bias} in 5 mM (a) and 50 mM (b) NaCl aqueous solution; c) ±0.15 V_{EC}; ±0.1 V_{bias} in 5 mM (d), 50 mM (e) and 500 mM (f) NaCl aqueous solution.

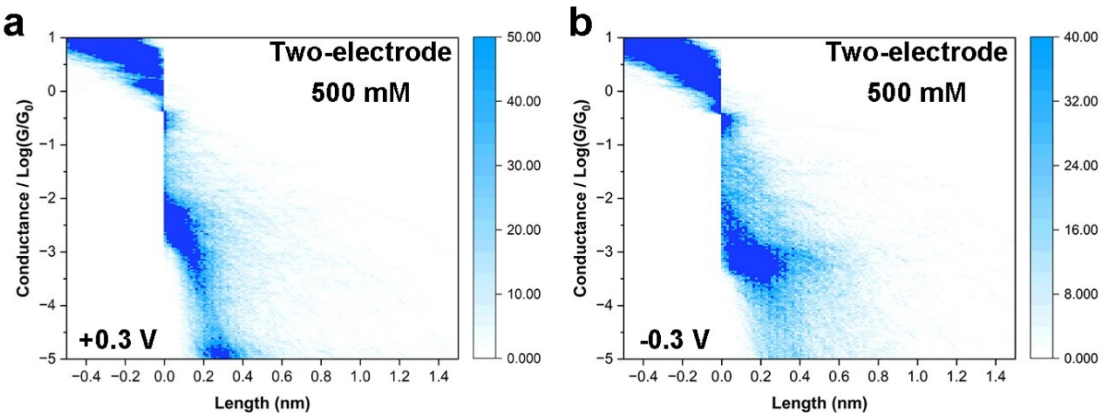

Figure S10. Two-dimensional conductance/distance histograms of HH. a) HH at bias of 0.3 V and b) -0.3 V in two-electrode system in 500 mM aqueous NaCl solution.

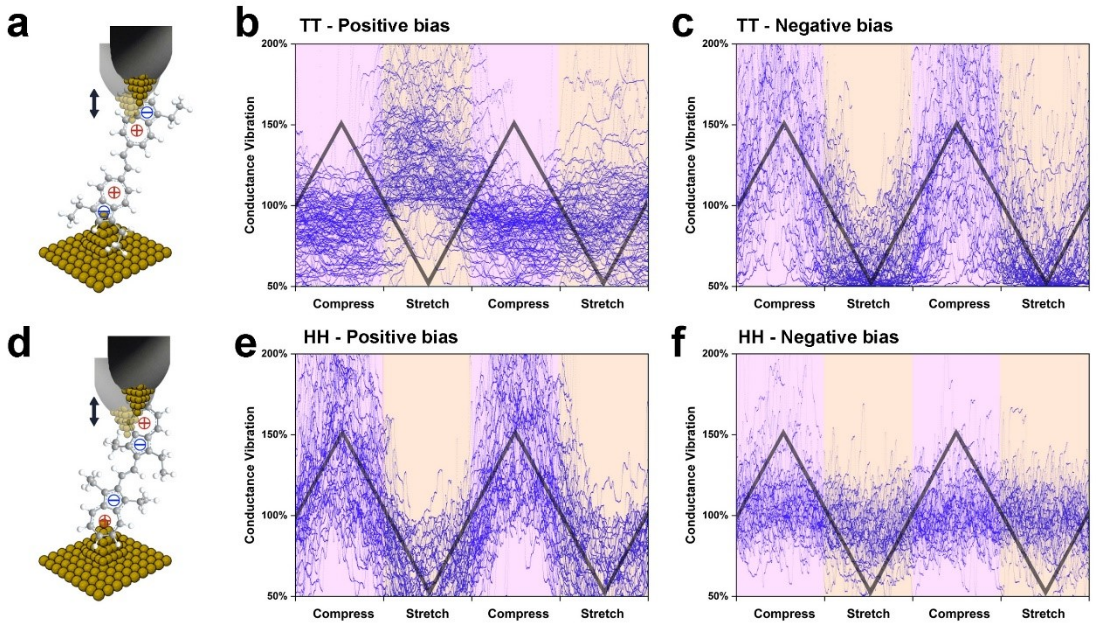

Figure S11. Mechanical modulation of molecule-electrode coupling. a) modulation of tip movement in TT single-molecule junctions at (b) 0.3 V and (c) -0.3 V bias in 500 mM electrolyte solution; d) modulation of tip movement in HH single-molecule junctions at 0.3 V (e) and -0.3 V (f) bias in 500 mM electrolytic solution. The conductance variation was normalized to the initial conductance of each single-molecule junction. The black solid line represents the triangular wave applied to the piezo.

For TT, at positive bias (0.3 V, **Fig. S11b**), the conductance was found to increase when the tip is moved farther from the substrate, especially during the first modulation cycle, and to decrease when the tip is moved closer. The trend suggests that a more distal anchoring site (farther from the molecule's center) on TT is favoured at positive bias. Conversely, at negative bias (-0.3 V, **Fig. S11c**), the conductance shows the opposite behaviour, with higher conductance at shorter distances and lower conductance at larger distances, implying a preference for a more proximal anchoring site at negative bias.

For HH, while we observed higher conductance at shorter distances at positive bias (0.3 V), the conductance change with distance at negative bias (-0.3 V) was more moderate (**Fig. S11f**). This observation suggests a more proximal anchoring position for HH at positive bias and a more distal binding site at negative bias, but the difference in binding position between the two orientations is smaller than for TT.

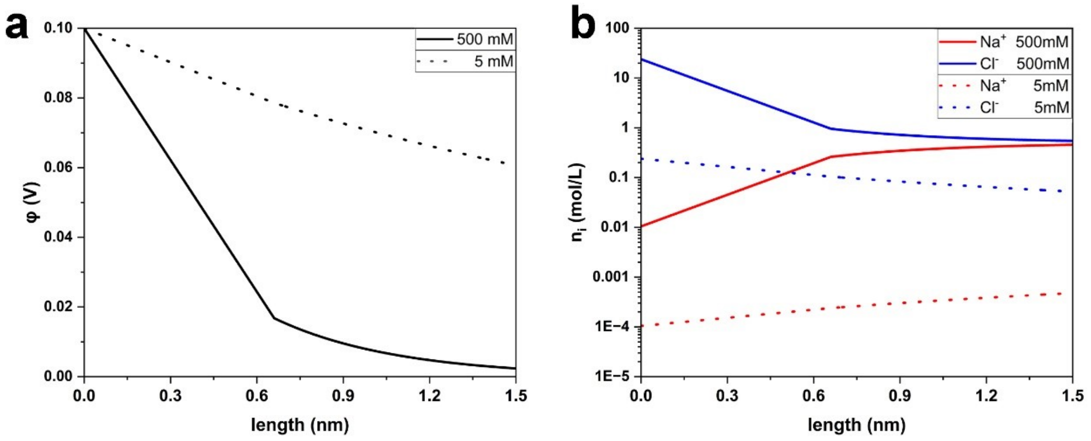

Figure S12. COMSOL simulation of a) potential profile and b) cation and anion density profiles at 500 mM and 5 mM electrolytic concentration and 0.1 V bias.

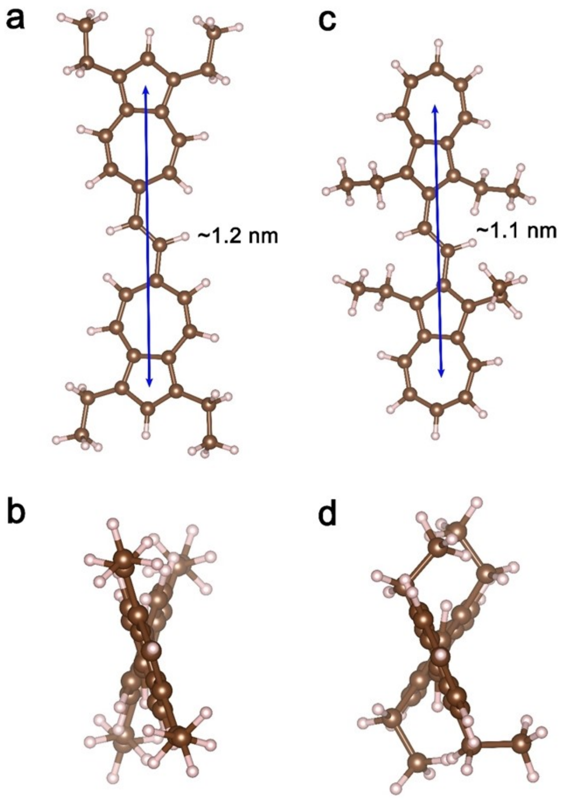

Figure S13. The optimized structures of TT and HH molecules at the DFT-B3LYP/6-311g(d) level. (a) Top view and (b) side view of the optimized TT molecule. (c) Top view and (d) side view of the optimized HH molecule. The spheres in brown and light coral represent C and H, respectively.

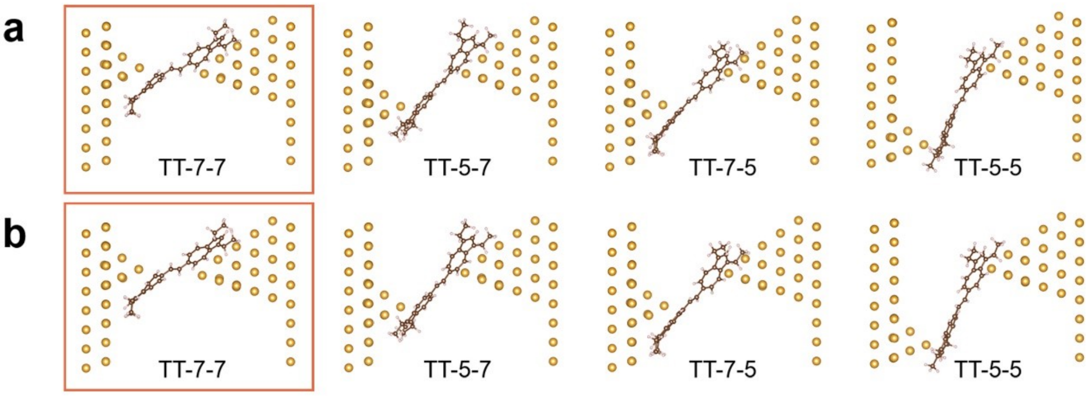

Figure S14. The optimized single-molecule junctions with different anchoring sites of azulene blocks in TT molecule on two electrodes in non-electrolytic solution at (a) 0.3 V and (b) -0.3 V. The balls in gold, brown, and light coral represent Au, C, and H, respectively. In the subsequent geometric optimizations (unless otherwise specified), the GGA-PBE functional is used, and a basis set of SZP for Au while DZP for C, H, Na^+, and Cl^- is employed. DFT-D3 semiempirical dispersion correction is applied to more accurately describe the Au-π electronic interaction

Table S1. The relative energy (in eV) of each optimized single-molecule junction with different anchoring sites of azulene blocks in TT molecule on two electrodes in non-electrolytic solution.

Bias Voltage	TT-7-7	TT-5-7	TT-7-5	TT-5-5
0.3 V	0	1.76	0.39	2.53
-0.3 V	0	1.38	0.34	2.59

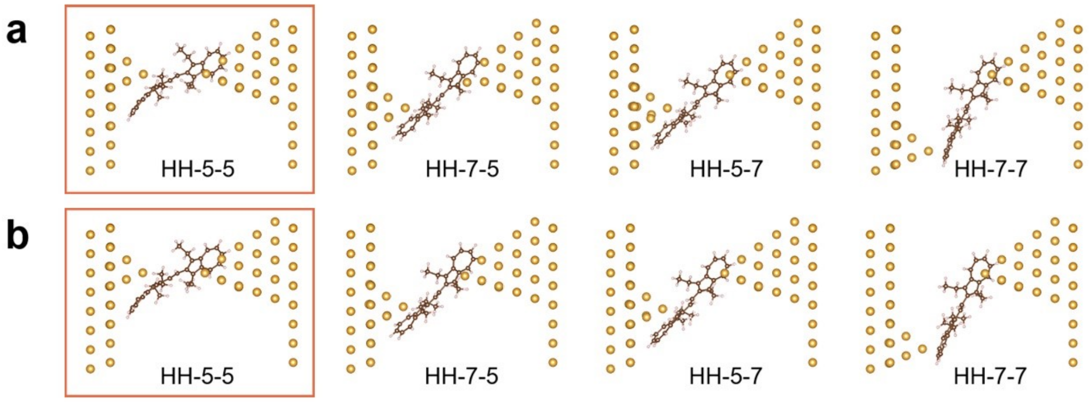

Figure S15. The optimized single-molecule junctions with different anchoring sites of the azulene blocks in the HH molecule on two electrodes in non-electrolytic solution at (a) 0.3 V and (b) -0.3 V. The spheres in gold, brown, and light coral represent Au, C, and H, respectively.

Table S2. The relative energy (in eV) of each optimized single-molecule junction with different anchoring sites of azulene blocks in the HH molecule on two electrodes in non-electrolytic solution.

Bias Voltage	HH-5-5	HH-7-5	HH-5-7	HH-7-7
0.3 V	0	0.82	0.82	2.18
-0.3 V	0	0.72	0.54	1.94

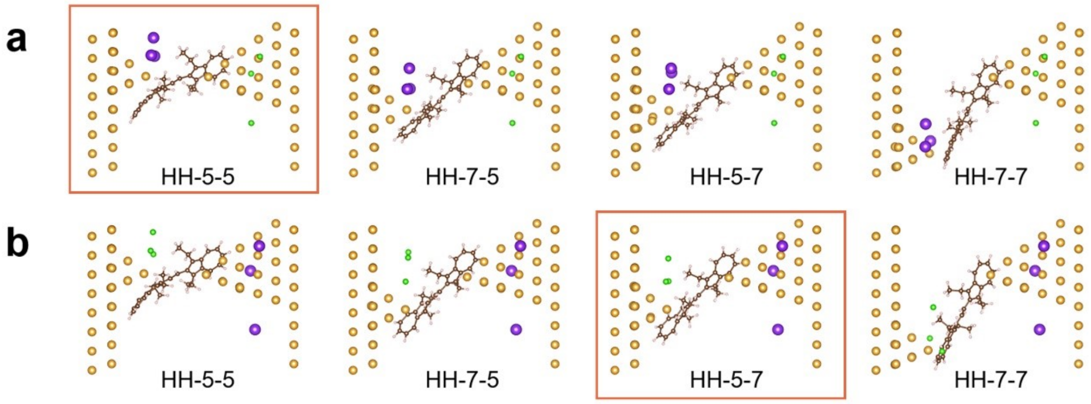

Figure S16. The optimized single-molecule junctions with different anchoring sites of azulene blocks in the HH molecule on two electrodes in 500 mM NaCl electrolytic solution at (a) 0.3 V and (b) -0.3 V. The spheres in gold, brown, light coral, green, and purple represent Au, C, H, Cl^-, and Na^+ respectively.

Table S3. The relative energy (in eV) of each optimized single-molecule junction with different anchoring sites of azulene blocks in the HH molecule on two electrodes in 500 mM NaCl electrolyte solution.

Bias Voltage	HH-5-5	HH-7-5	HH-5-7	HH-7-7
0.3 V	0	0.02	0.12	0.20
-0.3 V	0.39	0.38	0	1.17

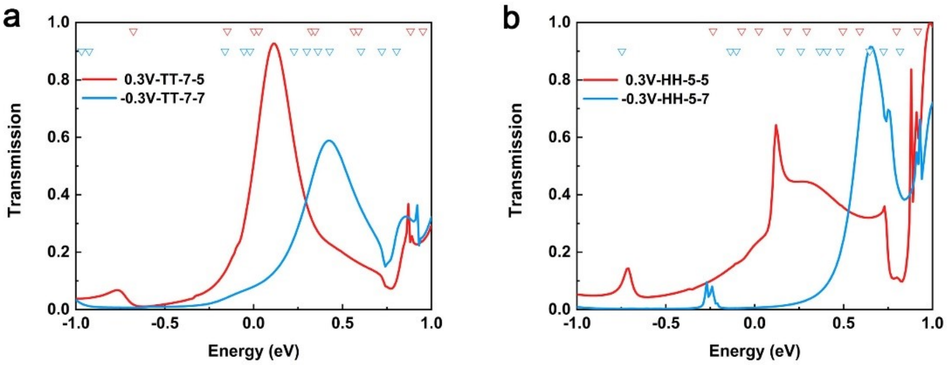

Figure S17. Transmission spectra of single-molecule junctions in 500 mM NaCl electrolyte solution at ±0.3 V for (a) TT and (b) HH. The triangles indicate the MPSH eigenvalues. The HSE06 functional is used in the transport calculations, and a basis set of SZP for Au, and DZP for C, H, Na^+, Cl^- is employed.

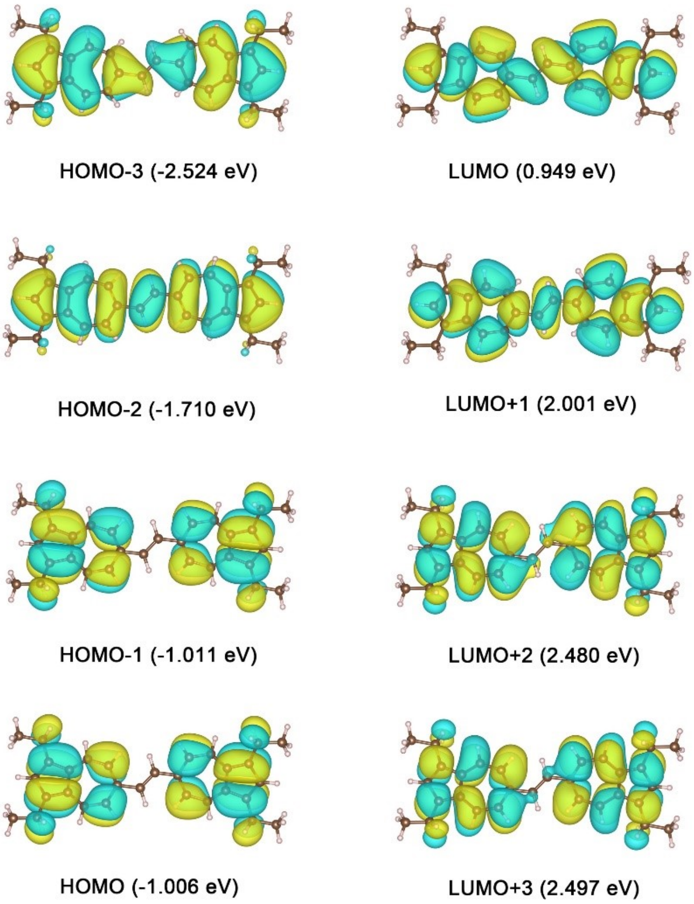

Figure S18. The frontier molecular orbitals for TT bare molecule. The isovalue is 0.01 $Bohr^{-3/2}$. Cyan and yellow denotes different phases of molecular orbitals.

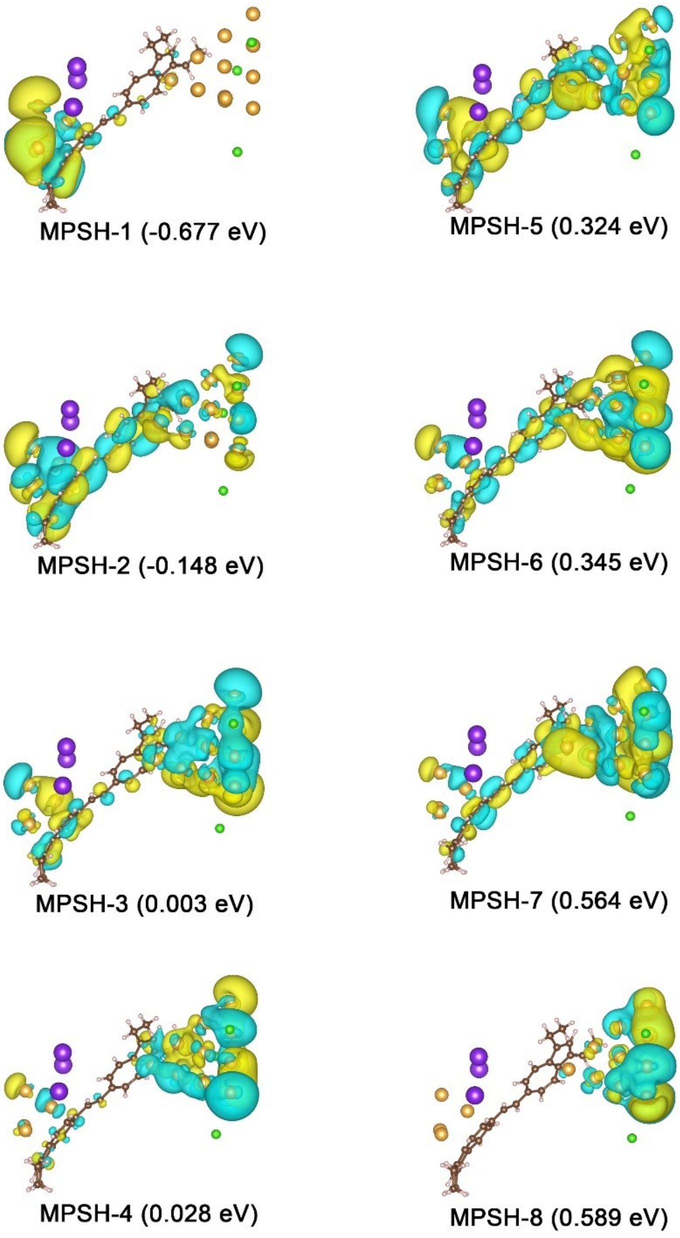

Figure S19. The MPSH states for TT in TT-7-5 single-molecule junction at 0.3 V. The isovalue is 0.01 Bohr$^{-3/2}$. Cyan and yellow denotes different phases of molecular orbitals. Purple and green represents Na^{+} and Cl^{-}.

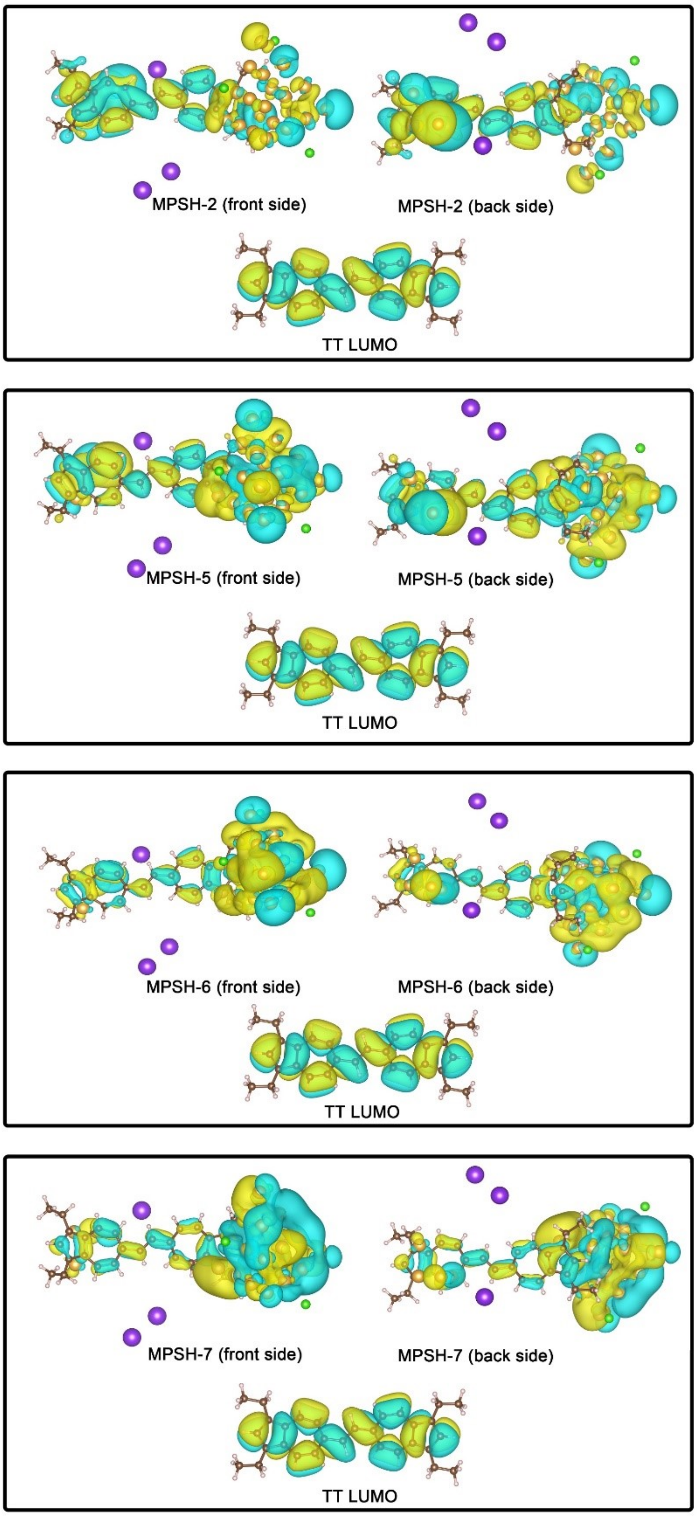

Figure S20. Assignments of the MPSH states to the bare molecular orbital for TT in TT-7-5 single-molecule junction at 0.3 V. The isovalue is 0.01 Bohr$^{-3/2}$. Cyan and yellow denotes different phases of molecular orbitals. Purple and green represents Na^+ and Cl^-.

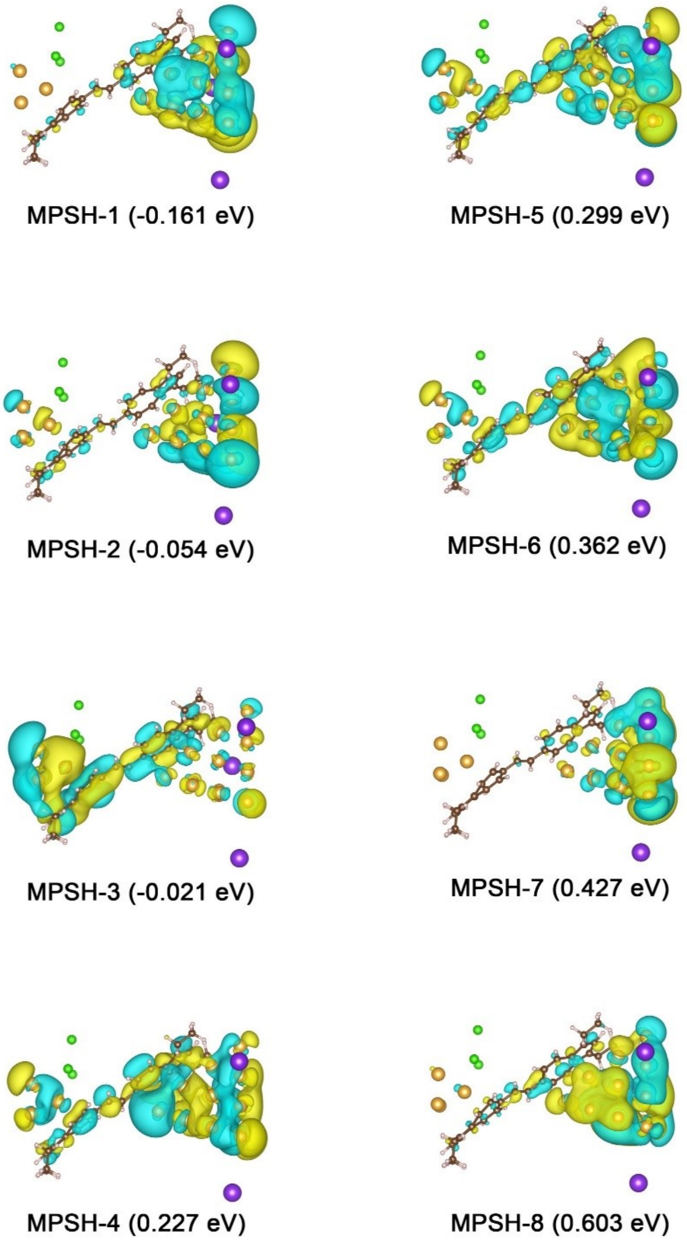

Figure S21. The MPSH states for TT in TT-7-7 single-molecule junction at -0.3 V. The isovalue is 0.01 Bohr$^{-3/2}$. Cyan and yellow denotes different phases of molecular orbitals. Purple and green represents Na^+ and Cl^-.

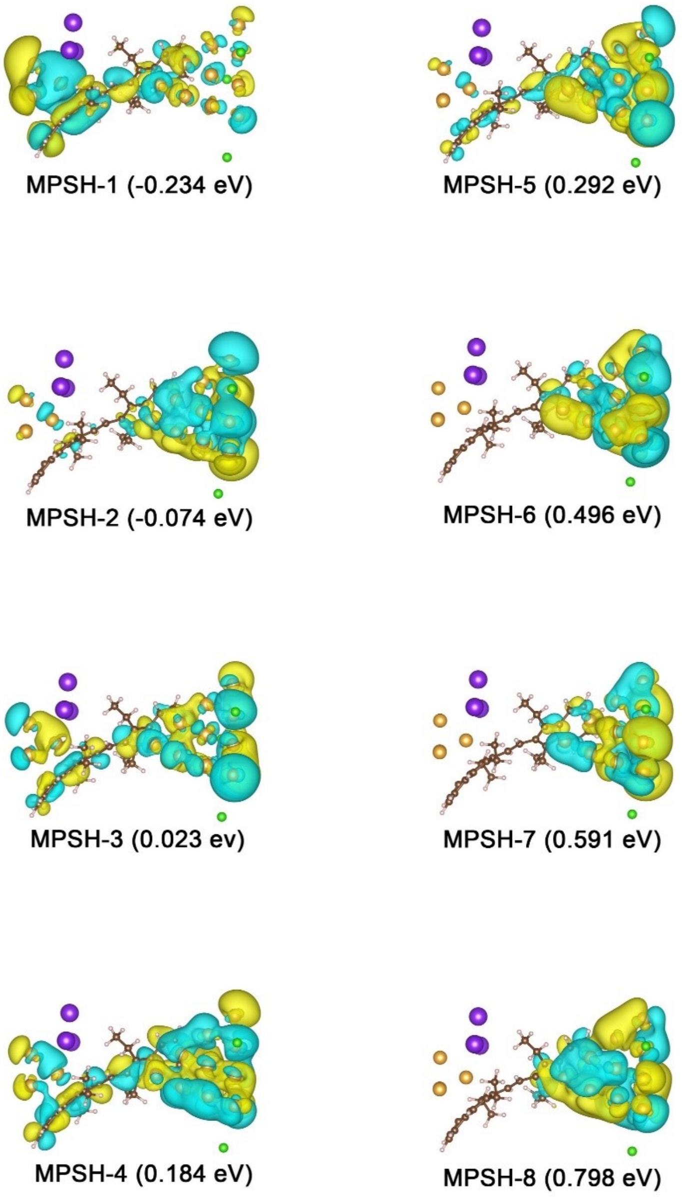

Figure S22. The MPSH states for HH in HH-5-5 single-molecule junction at 0.3 V. The isovalue is 0.01 Bohr$^{-3/2}$. Cyan and yellow denotes different phases of molecular orbitals. Purple and green represents Na^{+} and Cl^{-}.

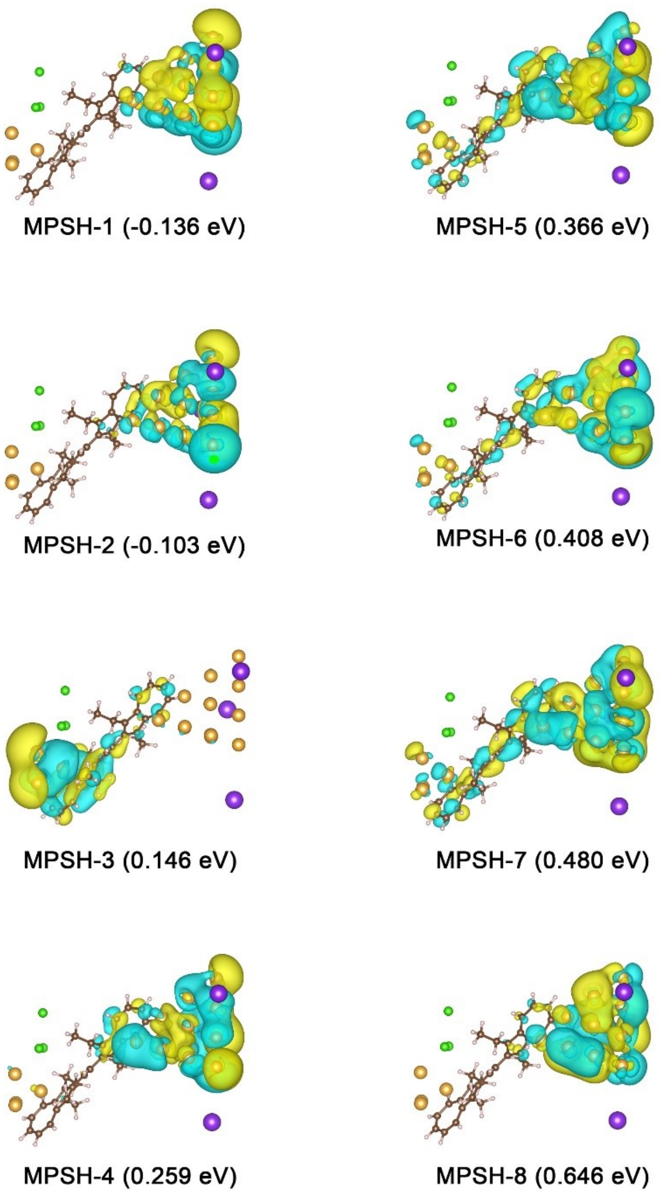

Figure S23. The MPSH states for HH in HH-5-7 single-molecule junction at -0.3 V. The isovalue is 0.01 Bohr$^{-3/2}$. Cyan and yellow denotes different phases of molecular orbitals. Purple and green represents Na^{+} and Cl^{-}.
